# Chromosome structure and DNA replication dynamics during the life cycle of the predatory bacterium *Bdellovibrio bacteriovorus*

**DOI:** 10.1093/femsre/fuad057

**Published:** 2023-10-03

**Authors:** Karolina Pląskowska, Jolanta Zakrzewska-Czerwińska

**Affiliations:** Department of Molecular Microbiology, Faculty of Biotechnology, University of Wrocław, ul. Joliot-Curie 14A, Wrocław, Poland; Department of Molecular Microbiology, Faculty of Biotechnology, University of Wrocław, ul. Joliot-Curie 14A, Wrocław, Poland

**Keywords:** bacterial life cycle, binary and nonbinary division, chromosome organization, initiation of chromosome replication, *oriC*, choreography of chromosome replication

## Abstract

*Bdellovibrio bacteriovorus*, an obligate predatory Gram-negative bacterium that proliferates inside and kills other Gram-negative bacteria, was discovered more than 60 years ago. However, we have only recently begun to understand the detailed cell biology of this proficient bacterial killer. *Bdellovibrio bacteriovorus* exhibits a peculiar life cycle and bimodal proliferation, and thus represents an attractive model for studying novel aspects of bacterial cell biology. The life cycle of *B. bacteriovorus* consists of two phases: a free-living nonreplicative attack phase and an intracellular reproductive phase. During the reproductive phase, *B. bacteriovorus* grows as an elongated cell and undergoes binary or nonbinary fission, depending on the prey size. In this review, we discuss: (1) how the chromosome structure of *B. bacteriovorus* is remodeled during its life cycle; (2) how its chromosome replication dynamics depends on the proliferation mode; (3) how the initiation of chromosome replication is controlled during the life cycle, and (4) how chromosome replication is spatiotemporally coordinated with the proliferation program.

## Teaser

A predatory bacterium, *Bdellovibrio bacteriovorus* possesses a tightly packed chromosome and exhibits a unique “asynchronous mode” of replication (re)initiation.

## Introduction

**Figure 1.**
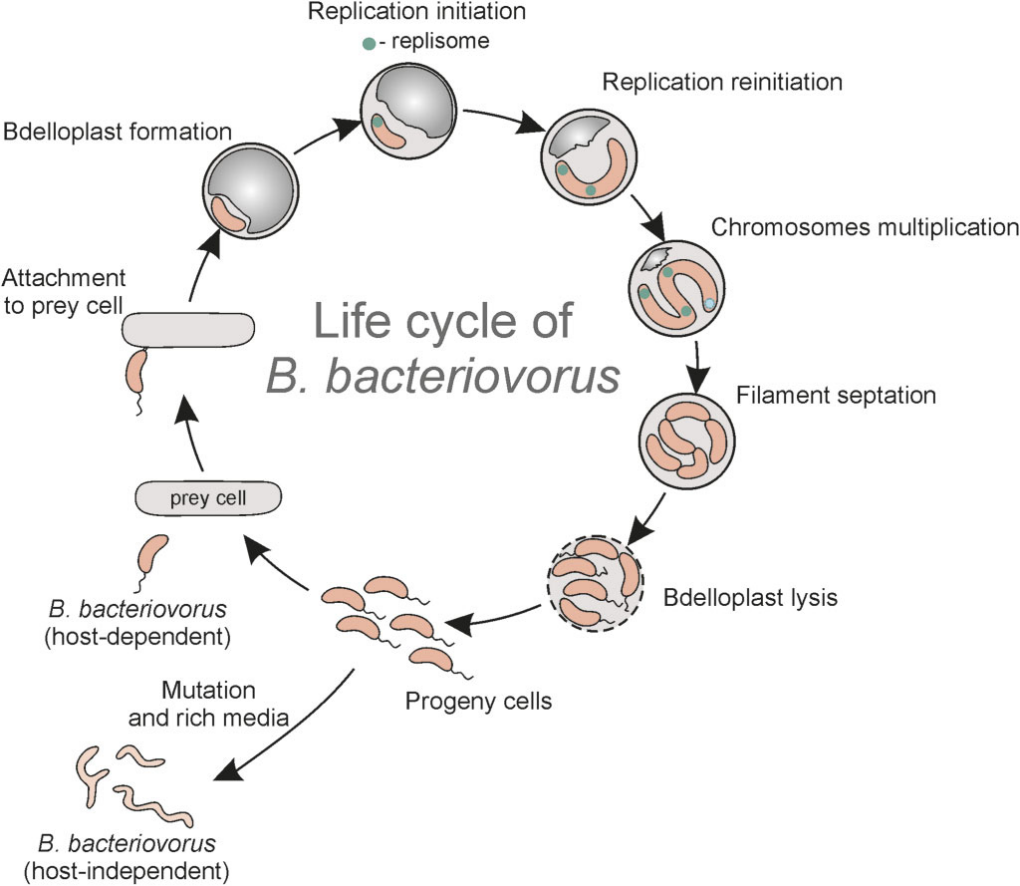
Predatory life cycle of *B. bacteriovorus*. The life cycle of *B. bacteriovorus* consists of an the attack phase and a the proliferation phase. During the attack phase, a free-swimming, mono-flagellated *B. bacteriovorus* cell searches for a prey cell and then attaches via its invasive pole to the prey’s outer membrane. The predatory cell then passes through the outer membrane and peptidoglycan layer into the prey’s periplasm. Using a plethora of lytic enzymes, it changes the prey’s cell shape (Lerner et al. 2012) and forms a bdelloplast (dead host cell). From this point, the proliferation phase begins. *B. bacteriovorus* digests the bdelloplast’s cytosolic contents and reuses simple compounds to build its own structures. It grows as a filament, inside which chromosome multiplication and segregation occur simultaneously. Chromosome replication is usually initiated at the invasive pole (green replisome), but initiation may occur at the opposite pole (blue replisome) in filaments dividing into five or more progeny cells (see text for details). When the prey cell’s resources are exhausted, the *B. bacteriovorus* filament synchronously septates and forms an odd or even number of unigenomic progeny cells. Finally, the mature daughter cells are released from the bdelloplast into the environment. Rarely occurring saprophytic variants of *B. bacteriovorus* can exhibit host-independent (HI) growth.

François Jacob stated that “the dream of a bacterium is to become two bacteria” (Jacob 1965). Most bacteria, including well-known model organisms like *Escherichia coli, Bacillus subtilis*, and *Caulobacter crescentus*, use binary division to produce two daughter cells from one parent cell. However, some so-called “nonclassical” bacteria use nonbinary fission, which is a more complex cell cycle mode wherein the chromosome replicates into three or more copies and the resulting multinucleoid elongated cell divides into three or more progeny cells. Examples of organisms in this group include Gram-positive bacteria, such as the antibiotic-producing *Streptomyces*, and the predatory Gram-negative bacterium, *Bdellovibrio bacteriovorus*, which exclusively proliferates inside other Gram-negative bacteria (Fig. 1). Recent work demonstrated that *B. bacteriovorus* can reproduce through nonbinary or binary fission (Pląskowska et al. 2023). The transition between those two modes is correlated with the size of the prey (see Figs 3 and 4): predation on small cells, like *Proteus mirabilis*, results in the formation of two progeny cells, while predation on larger prey cells results in the formation of three or more progeny cells (Pląskowska et al. 2023, Santin et al. 2023).

The life cycle of host-dependent (HD) *B. bacteriovorus* consists of two phases: a free-living nonreplicative attack phase and an intracellular reproductive phase (for details see Fig. 1; for reviews, see Sockett 2009, Rotem et al. 2014, Negus et al. 2017, Caulton and Lovering 2020, Laloux 2020, Lai et al. 2023). During the attack phase, the *B. bacteriovorus* cell is asymmetrically arranged with a flagellum at one cell pole and a pili at the other (the invasive pole). The flagellum facilitates prey-searching movement (Kaplan et al. 2023) and the pili are required for the predation (Evans et al. 2007, Kaplan et al. 2023). During the reproductive phase, *B. bacteriovorus* grows as an elongated cell (called a filament) in the periplasm of its prey. When the host’s nutrients are exhausted, the filament, which contains two or more chromosomes, septates into the appropriate even or odd number of unigenomic progeny cells.

During the attack phase, the predatory cells are quite small (0.2–0.5 µm wide and 0.5–1.4 µm long). However, unlike most other obligatory intracellular bacteria, *B. bacteriovorus* has not undergone a significant reduction of genome size, and thus possesses a relatively large chromosome (3.8 Mbp) (Rendulic et al. 2004).

Wild-type *B. bacteriovorus* is an obligate predator, but rare saprophytic mutants can grow in the absence of prey and thus act as host-independent (HI) mutants (Figs 1 and 4) (Cotter and Thomashow 1992a). These HI mutants occur as irregularly shaped filaments (Figs 1 and 4) and grow very slowly on rich media (Cotter and Thomashow 1992a, b).

In this review, we present the organization of the *B. bacteriovorus* chromosome, explore how it changes during the life cycle, discuss the choreography of chromosome replication during the binary and nonbinary proliferation of this predatory bacterium, and finally compare the replication choreography of *B. bacteriovorus* with those of bacteria that proliferate by either binary or nonbinary modes.

## The chromosome of *B. bacteriovorus* must be extremely compacted

The observation that *B. bacteriovorus* possesses a relatively large chromosome raises the following important questions: (1) Why does an obligate predator that proliferates in other bacteria have so many genes? (2) How is such a large chromosome compacted in a tiny cell? and (3) How is the chromosome structure synchronized with the unusual life cycle of *B. bacteriovorus*?

### Why does an obligate predator that proliferates in other bacteria have so many genes?

**Figure 2.**
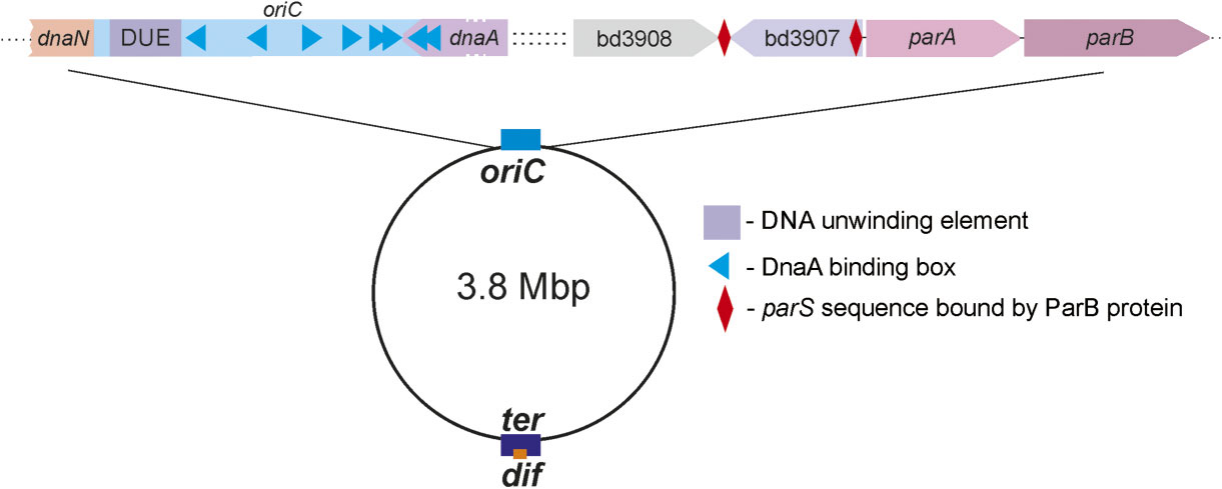
Localization of the origin of chromosome replication *(oriC)*, the *parA* and *parB* genes, and the *parS* sequence on the *B. bacteriovorus* chromosome. The directions of triangles within the *oriC* region represent the orientations of particular DnaA boxes bound by the DnaA protein (Makowski et al. 2016). The diamond represents the palindromic *parS* sequence bound by the ParB protein (Kaljević et al. 2023). The distance between the *dnaA* (encoding the DnaA protein) and *bd3908* (encoding a putative rRNA methyltransferase) genes is 6466 bp. *Ter* and *dif* indicate the replication termination region and the site that ensures the resolution of chromosome dimers, respectively (Carnoy and Roten 2009, Kaljević et al. 2021). The figure is not drawn to scale.

*B. bacteriovorus* HD100 resembles most other bacteria in possessing a single covalently closed chromosome. However, this chromosome contains many more genes (3584 predicted ORFs; 3.8 Mbp) (Rendulic et al. 2004) (Fig. 2) than other obligate intracellular parasites, such as *Chlamydia* (894–1052 ORFs; 1.0–2.0 Mbp) (Stephens et al. 1998, Read et al. 2000, Sigalova et al. 2019, Stelzner et al. 2023) and *Rickettsia* (872–1512 ORFs; 1.2–1.3 Mpb) (McLeod et al. 2004, Blanc et al. 2007, Gillespie et al. 2008, McGinn and Lamason 2021). This likely reflects that, compared to these other intracellular pathogens, *B. bacteriovorus* exhibits a more complex life cycle (see Fig. 1) and acts as an obligate predator of other Gram-negative bacteria. The *B. bacteriovorus* genome contains many diverse genes that are essential for its predatory behaviors, which include chemotaxis, attachment to the prey cell, entry into the prey’s periplasm, adaptation to the host bacterium and, most importantly, digestion of the bdelloplast content. To feed on its prey, *B. bacteriovorus* requires a wide repertoire of enzymes that can degrade different types of macromolecules. Indeed, the *B. bacteriovorus* genome encodes a plethora of hydrolytic enzymes (~300) (Rendulic et al. 2004) that degrade DNA, RNA, proteins, lipids, polysaccharides, and so on. The products of hydrolysis are reused for the predator’s growth and replication, and up to 80% of degraded host nucleic acids are incorporated into the DNA of *B. bacteriovorus* (Matin and Rittenberg 1972). *B. bacteriovorus* cannot endogenously synthesize certain amino acids; instead, these critical components presumably must be obtained from the products of prey protein hydrolysis. Therefore, *B. bacteriovorus* possesses a large repertoire of transporters (more than 100) for transporting amino acids, peptides, amines, and even nucleotides, which are rarely transported in bacteria (Barabote et al. 2007). The transporters are also essential for HI mutants growing on media rich in amino acids. The *B. bacteriovorus* genome further contains many genes encoding hypothetical proteins (1207 ORFs, comprising one-third of the total ORFs) (Rendulic et al. 2004). Additional work is needed to assign their biological roles.

In summary, unlike the genomes of other obligate intracellular parasites, that of *B. bacteriovorus* is surprisingly large and contains an enormous number of genes encoding enzymes and transporters, that are indispensable for the particular life cycle of this predatory bacterium.

### How is such a large chromosome compacted in a tiny cell?

**Figure 3.**
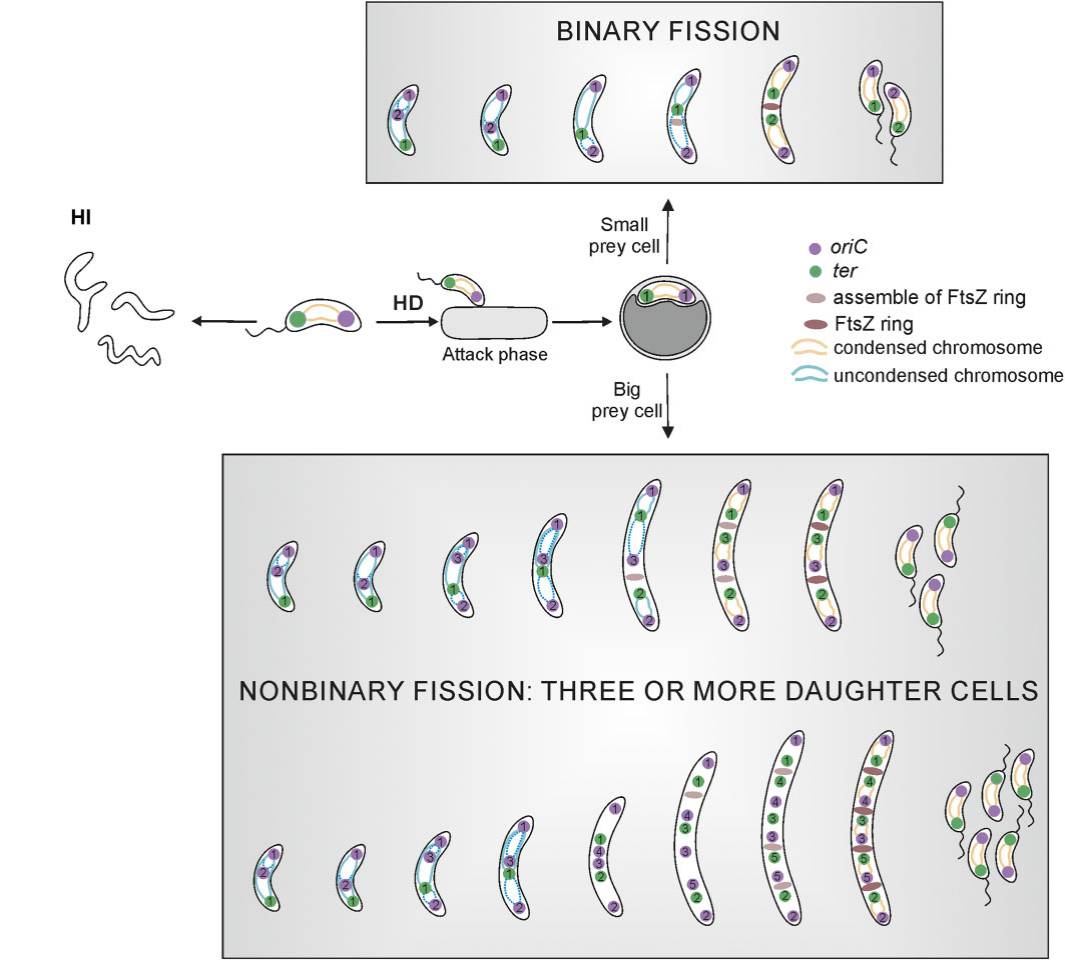
Asynchronous choreography of the initiation of chromosome replication in *B. bacteriovorus* dividing by binary or nonbinary fission. During the attack phase, the *B. bacteriovorus* chromosome is highly compacted (yellow, solid line) and longitudinally arranged, with the *oriC* (violet) and *ter* (green) regions positioned near the invasive and flagellated poles, respectively. *B. bacteriovorus* undergoes either binary fission (in small prey cells) or nonbinary fission (in big prey cells). During the adaptation phase, which follows the invasion of prey, the chromosome is still tightly compacted. At the beginning of the proliferation phase, chromosome replication is initiated at the invasive pole regardless of the cell division mode, and the chromosome becomes decondensed (blue, solid line). During ongoing replication (dark blue dashed line), one of the duplicated *oriC* regions is segregated to the former flagellar pole and then the *ter* region relocates to mid-cell. In both binary and nonbinary dividing cells, Z-ring assembly begins prior to the termination of DNA replication. In a binary-dividing cell, DNA replication is terminated at the mid-cell. In filaments dividing by nonbinary fission, the second round of DNA replication is reinitiated from the chromosome located at the invasive pole; after segregation, one of the newly synthesized *oriC* copies (the third copy) colocalizes with the first *ter* in the middle of the filament. The (re)initiation of further rounds of chromosome multiplication also takes place at the invasive pole (see text for details), and the copy number of *oriC* increases sequentially. At the final stage of replication, the Z-rings are asynchronously assembled and the filament is synchronously divided into an odd or even number of progeny cells, each of which contains a compacted and polarized chromosome (adapted from Kaljević et al. 2021, Pląskowska et al. 2023).

During the attack phase, no DNA synthesis occurs, most genes are downregulated (Karunker et al. 2013), and the predator utilizes ATP mainly to move in search of prey. In this phase of the life cycle, the predator cell can be as small as 0.3 µm wide and 0.8 µm long (Stolp and Petzold 1962, Laloux 2020). Consequently, the chromosome must be more than 1500 times shorter than the naked (devoid of proteins) linear DNA (∼1.3 mm). The chromosome is so tightly compacted within the small cell that it cannot be penetrated even by small monomeric fluorescent proteins (Kaljević et al. 2021). As observed for the chromosomes of *C. crescentus* and *Vibrio cholerae*, that of *B. bacteriovorus* is longitudinally arranged, with the origin of chromosome replication (*oriC*) and the terminus of replication (*ter*) positioned near the invasive and flagellated poles, respectively (Fig. 3) (Kaljević et al. 2021). Transmission electron microscope and cryoelectron tomography revealed that the chromosome exhibits a spiral architecture and is organized in two twisted strands along the longitudinal axis of the cell (Butan et al. 2011, Kaplan et al. 2023). This spiral organization presumably facilitates the efficient compaction of the *B. bacteriovorus* chromosome. Despite these observations, however, relatively little is known about how the *B. bacteriovorus* chromosome is organized.

Bacterial chromosomes are compacted by various processes and factors, including molecular crowding, depletion forces, DNA supercoiling (topoisomerases), condensins (SMC, MukB, and Mks), and small basic nucleoid-associated proteins (NAPs) (Badrinarayanan et al. 2015, Verma et al. 2019, Dame et al. 2020, Norris et al. 2022). The chromosomes of model bacteria, mainly *E. coli, B. subtilis*, and *C. crescentus*, exhibit a multilevel hierarchical organization that ranges from large-scale macrodomains (in the Mbp range) to smaller (∼10 kb) topologically independent microdomains. The organization of bacterial chromosomes is profoundly influenced by diverse sets of NAPs that compact chromosomes by bending (HU, IHF, Fis, and Dps), bridging (H-NS), or wrapping (Lrp) the DNA (Luijsterburg et al. 2006, Dillon and Dorman 2010, Wang et al. 2011, Prieto et al. 2012, Badrinarayanan et al. 2015, Hołówka and Zakrzewska-Czerwińska 2020, Lioy et al. 2021). Genes encoding homologs of classic bacterial NAPs, including HU (Bd2104), IHF (Bd1639), Fis (Bd0039), and Dps (Bd2620), have been identified within the *B. bacteriovorus* genome (The Bdellovibrio viewer: *B. bacteriovorus* transcriptome 2023). Additionally, *B. bacteriovorus* was recently shown to encode two histones (Bd0055 and Bd3044) that contain putative histone-fold domains (Hocher et al. 2023). *In vitro* studies revealed that, unlike a “classical” histone, the Bd0055 protein does not wrap DNA around itself but instead completely coats linear DNA by binding end-to-end and thereby forming a nucleohistone filament (Hocher et al. 2023, Hu et al. 2023). The Bd0055 gene is highly expressed mainly during the proliferation phase and ranks among the top 6% of genes with the highest expression level (Hocher et al. 2023, The Bdellovibrio viewer: *B. bacteriovorus* transcriptome 2023). Importantly, the Bd0055 and Bd3044 genes are essential throughout the *B. bacteriovorus* life cycle, as their deletion has been shown to be lethal. However, their functions and impact on DNA organization have yet to be elucidated *in vivo*. Prior to this work, histones had not been identified in bacteria were commonly believed to be exclusive to eukaryotes and archaea.

Similar to the situation in other bacteria, the global structure of the *B. bacteriovorus* chromosome appears to be maintained by condensins, such as SMC, which spatially and dynamically organizes bacterial chromosomes by extruding DNA into large loops. In the *B. bacteriovorus* genome, the Bd1158 gene was identified to encode the SMC protein (The Bdellovibrio viewer: *B. bacteriovorus* transcriptome 2023). In addition to typical bacterial topoisomerases, such as TopA (Bd0964), Top II (Bd2865), and Top IV (Bd0005), the *B. bacteriovorus* genome encodes topoisomerase VI (Bd2864) (The Bdellovibrio viewer: *B. bacteriovorus* transcriptome 2023), which is an archaeal- and plant-type topoisomerase (Forterre et al. 2007).

Thus, a diverse range of proteins, including unusual ones such as histones and an archaeal/plant topoisomerase, is presumably involved in the chromosomal architecture of *B. bacteriovorus*. Notably, chromosomes must also be extensively compacted during sporulation in *B. subtilis* and *Streptomyces* to fit into the tiny spore compartment (Khanna et al. 2020, Szafran et al. 2020). However, our understanding of chromosome organization during sporulation also remains incomplete.

### How is the chromosome structure synchronized with the unusual life cycle of *B. bacteriovorus*?

**Figure 4.**
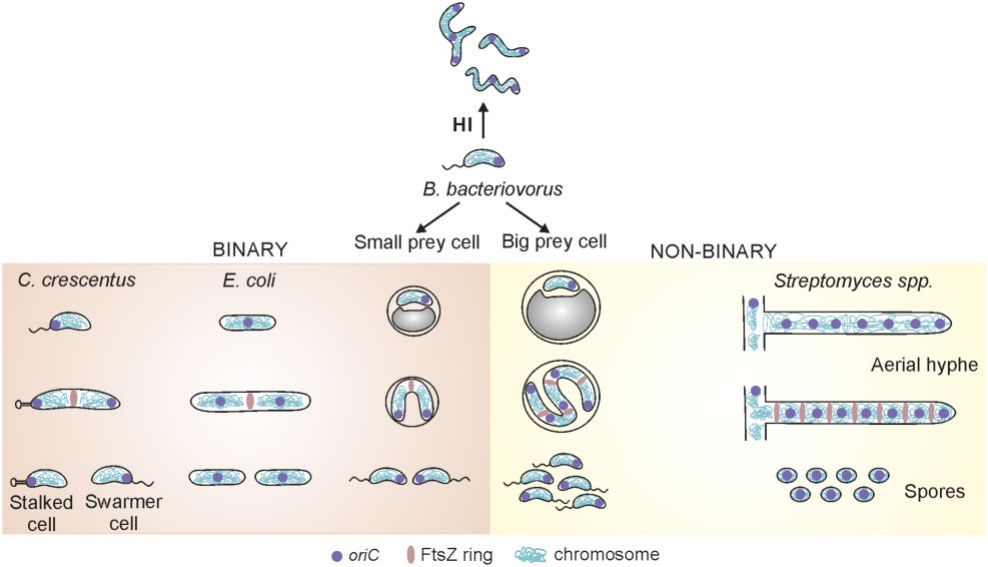
Localization of *oriC* region(s) in bacteria undergoing binary and/or nonbinary fission (see text for details).

In the attack phase, the *B. bacteriovorus* chromosome is highly compacted; during the proliferation phase, the DNA must be accessible to proteins involved in essential cellular processes, such as DNA replication, chromosome segregation, and transcription (Fig. 3). Unlike the situation in eukaryotic organisms, these processes occur simultaneously in bacteria. Consequently, the *B. bacteriovorus* chromosome undergoes dynamic morphological changes during proliferation, particularly at the beginning of this phase. After chromosome replication is initiated, the chromosome decondenses and remains in this state until the elongated cell is ready to divide (Kaljević et al. 2021). The decondensation of the chromosome has been suggested to be triggered first by replication initiation and further stimulated by the progression of replication forks (Kaljević et al. 2021). Importantly, the length of the elongated cell can exceed 20 times its initial length. Daughter chromosomes occupy most of the filament and do not show obvious signs of compaction (Kaljević et al. 2021). Before the multiple filament constrictions undergo closure, the chromosomes are reorganized into compact structures to ensure that each tiny daughter cell receives one copy. This resembles the sporulation of the nonbinary dividing bacterium, *Streptomyces*, when dozens of chromosomes must be segregated and condensed to ensure the proper formation of unigenomic spores (see Fig. 4) (Jakimowicz and van Wezel 2012).

Notably, in bacteria, the composition and levels of NAPs can change during the life cycle, leading to global alterations in the chromosome (Amemiya et al. 2021). NAPs, apart from their architectural functions, also crucially contribute to cellular processes, such as DNA replication (e.g. HU, IHF, and Fis) and transcription (e.g. H-NS) (Hołówka and Zakrzewska-Czerwińska 2020). Therefore, it is plausible that, during the *B. bacteriovorus* life cycle, certain NAPs may not only shape the chromosome architecture but also regulate DNA replication and modulate the transcriptional profile of the cell.

In summary, the *B. bacteriovorus* chromosome undergoes dramatic structural changes as the predatory cell progresses through its life cycle. Further research is needed to reveal the relevant proteins and mechanisms in the chromosomal compaction of *B. bacteriovorus*. The application of techniques such as chromosome conformation capture (Hi-C) and quantitative high-resolution microscopy can provide insights into the three-dimensional structure and life cycle-related changes of the *B. bacteriovorus* chromosome.

## Big and well-fed hosts are the predator’s dream home

*B. bacteriovorus* exhibits an extraordinary ability to adapt to various Gram-negative hosts (Markelova 2010, Richards et al. 2012, Loozen et al. 2015, Shatzkes et al. 2016, Pląskowska et al. 2023). *B. bacteriovorus* can proliferate within a prey cell of any size, but the prey cell size is important: In a small prey like *P. mirabilis, B. bacteriovorus* forms only two daughter cells; in a large prey like exponentially growing *E. coli*, it can produce dozens of offspring (Kaljević et al. 2021, Pląskowska et al. 2023, Santin et al. 2023) (Figs 3 and 4). Interestingly, while the minimum number of formed daughter cells is obvious (two), the maximum number has not been conclusively determined. Up to 17 offspring were observed (Santin et al. 2023) in *E. coli*, but it remains possible that an even larger prey could yield an even greater number of daughter cells. Stolp observed 20–30 cells in *Aquaspirillum serpens* (Stolp 1967, Jurkevitch 2006).

The proliferation phase of *B. bacteriovorus* must be precisely adjusted to the size and “quality” of the prey cell. Recent studies demonstrated that the duration of *B. bacteriovorus* proliferation depends on: (1) the size of the prey cell, which determines the number of predator offspring (Pląskowska et al. 2023, Santin et al. 2023); and (2) the nutritional quality of the prey cell, which affects the rate of predator elongation (Santin et al. 2023). It is worth noting that, in contrast to the duration of later cell cycle stages (e.g. cell division), the duration of DNA replication, known as the S-phase, is primarily influenced by the size and nutritional quality of the prey cell (Pląskowska et al. 2023, Santin et al. 2023). In larger prey cells, more copies of the chromosome are synthesized, resulting in a longer period of DNA synthesis (see below). In “poorly nourished” prey cells (grown on minimal medium prior infection), the rate of *B. bacteriovorus* DNA synthesis is lower (and the S-phase is longer) than that in “well-fed” prey cells. Therefore, the spatiotemporal coordination of basic cell cycle processes, particularly that of chromosome replication with the proliferation program of *B. bacteriovorus*, is necessary for the effective exploitation of resources and space within an inhabited prey cell (Santin et al. 2023).

Rarely, *B. bacteriovorus* requires two independent prey cells to complete its life cycle. In this scenario, the undivided filament exits the bdelloplast and enters the second prey cell. After replication is completed, the filament undergoes septation, and the progeny cells leave the second bdelloplast. The noncanonical life cycle may be attributed to a limited assessment of the prey’s resources and/or size (Makowski et al. 2019).

### Choreography of chromosome replication during the reproductive phase

DNA replication is a complex and energy-consuming process that requires precise control. Across the three domains of life, chromosome replication is largely regulated at the initiation step, as a means to prevent unnecessary energy loss (Boye et al. 2000, Sclafani and Holzen 2007, Zakrzewska-Czerwińska et al. 2007). In *B. bacteriovorus*, chromosome replication occurs exclusively during the intracellular proliferation phase. Thus, the initiation step must be strictly controlled to ensure that DNA replication commences at an appropriate location and time.

#### (Re)initiation of chromosome replication

The key elements involved in the initiation of chromosome replication in *B. bacteriovorus*, namely the *oriC* region and the replication initiator protein, DnaA, have been characterized (Makowski et al. 2016). The *oriC* region of *B. bacteriovorus* exhibits a typical eubacterial *oriC* organization comprising DnaA protein binding motifs and an AT-rich DNA unwinding element (DUE) (Makowski et al. 2016) (Fig. 2). Similar to the situation in other bacteria, binding of the *B. bacteriovorus* DnaA protein to *oriC* triggers this region to unwind (Makowski et al. 2016) and thereby provide the entry site for a multiprotein replisome machinery that consists of a helicase, an RNA primase, and a DNA polymerase III holoenzyme (Leonard and Grimwade 2015, Wolański et al. 2015).

The initiation of bacterial chromosome replication is mainly regulated by controlling the activity and availability of *oriC* and/or DnaA. However, researchers have not yet identified any regulator of chromosome replication initiation in *B. bacteriovorus*. The activity of *B. bacteriovorus* DnaA presumably depends on its nucleotide-bound state (ATP versus ADP), as seen in other bacteria (Keyamura et al. 2007). RNA-seq analysis (Karunker et al. 2013) indicated that *dnaA* and other genes of the *Bdellovibrio* transcriptome involved in chromosome replication are expressed in the proliferation phase but nearly or completely silenced in the attack phase. This gene silencing may reflect the tight compaction of the chromosome during the attack phase, which is likely to block access to the *oriC* region and promoters, including those of genes involved in chromosome replication.

Chromosome replication does not begin immediately after bdelloplast formation, indicating that *B. bacteriovorus* requires some time to adapt to the prey environment (periplasm). The chromosome must first undergo decompaction to enable various DNA transactions, such as DNA replication and transcription. Real-time microscopic observation using fluorescent reporter strains of *B. bacteriovorus* (Makowski et al. 2019, Pląskowska et al. 2023) demonstrated that the first replisome appears several dozen minutes (∼45 min) after bdelloplast formation. However, there are exceptions: when the daughter cell leaves the bdelloplast and immediately attacks the next prey, chromosome replication starts earlier (the replisome appears ∼23 min after bdelloplast formation). This suggests that certain protein(s) involved in chromosome replication, particularly DnaA, are not fully degraded (Makowski et al. 2019). We cannot exclude the possibility that, as seen in *C. crescentus*, the DnaA protein undergoes specific proteolysis during the transition from the growth phase to the attack phase. However, a specific protease like Lon, which was found in *C. crescentus* (Jonas et al. 2013), has not yet been identified in *B. bacteriovorus*.

In *B. bacteriovorus*, the first round of chromosome replication is initiated at the invasive pole (Figs 1 and 3). The assembly of replisomes and the presence of the ParB complex (Kaljević et al. 2021, Pląskowska et al. 2023) at the cell pole suggest that, similar to the situations in other asymmetric bacterial cells (e.g. *C. crescentus* and *V. cholerae*) (Bowman et al. 2008, Yamaichi et al. 2012), the *B. bacteriovorus* chromosome is likely anchored to the cell pole (presumably through the *oriC* region) via one or more pole-localized proteins. It has been postulated that in *B. bacteriovorus*, the coiled-coil tropomyosin-like protein, DivIVA, together with the filament-forming protein, bactofilin (Laloux 2020, Milner et al. 2020), may be involved in maintaining cell polarity. In apically extending cells, such as *Corynebacterium, Mycobacterium*, and *Streptomyces*, DivIVA is the main component of the polar complex (Flärdh et al. 2012, Donovan and Bramkamp 2014). We cannot exclude the possibility that DivIVA and/or bactofilin are targets for yet unidentified proteins involved in anchoring the *B. bacteriovorus* chromosome at the cell pole. Overall, it remains unknown how polarity is achieved in this predatory bacterium.

Observation of further replication rounds has indicated that reinitiation(s) usually occurs at the invasive pole. Thus, the firing of subsequent replication rounds is an asynchronous process in which DNA replication is sequentially reinitiated from the chromosome located at the invasive pole, rather than from other replicated chromosomes. In long filaments (from which five or more progeny cells are formed), however, DNA synthesis may be initiated at the opposite pole in the late stages of chromosome multiplication (Fig. 1). In this case, the former flagellar pole must undergo extensive reconstruction to become an invasive pole that anchors a daughter chromosome, which can then undergo initiation of DNA synthesis. It is difficult to undertake microscopic observation of later stages of chromosome multiplication in filaments dividing into more than five progeny cells, because the long filaments are frequently twisted in the prey (Makowski et al. 2019, Pląskowska et al. 2023).

In conclusion, the “asynchronous mode” of replication reinitiation explains why filaments can contain an odd or even number of chromosome copies. The (re)initiation of DNA replication from the chromosome anchored to the invasive pole presumably ensures the precise spatial control of DNA replication in *B. bacteriovorus*, such that the initiation step is a crucial cell cycle checkpoint in this bacterium. Under nutritional stress (when the prey’s resources are exhausted), it seems reasonable to assume that one or more regulatory mechanisms) would be activated to prevent the next initiation of DNA replication and control further proliferation of the filament. In *C. crescentus*, the global transcription factor, CtrA, inhibits replication initiation by binding to the *oriC* region and additionally controls the expression of genes encoding regulators involved in cell cycle progression (Ryan et al. 2002). However, no regulatory mechanism(s) capable of preventing or triggering replication (re)initiation has yet been reported in *B. bacteriovorus*. Furthermore, it has been postulated that, during the adaptation phase, one or more specific cues reflecting prey quality will modulate chromosome replication and the subsequent steps of *B. bacteriovorus* proliferation (Rotem et al. 2015). Further studies are required to identify such cues and their targets, which would represent a cell-cycle checkpoint that would be expected to be universal across different prey cell types.

#### Chromosome multiplication

The spatiotemporal choreography of the next step in chromosome replication (i.e. elongation) depends on the proliferation mode and number of offspring (Fig. 3). *Bdellovibrio bacteriovorus* cells that divide by binary fission provide a simple model for studying chromosome replication dynamics (Fig. 3): From observation of *B. bacteriovorus* dividing into two daughter cells, the rate of DNA synthesis can be determined. Based on the C-period (the time required to replicate the *B. bacteriovorus* chromosome; e.g. 144 min for cells growing in *P. mirabilis*) (Pląskowska et al. 2023) and genome size (3.8 Mbp) (Rendulic et al. 2004), the DNA synthesis rate is estimated to be around 220 nucleotides per second (nt/s), which is approximately three to four times slower than that of *E. coli* (600–1000 nt/s) (Fijalkowska et al. 2012). This reduced replication rate might reflect the limited availability of nutrients, particularly nucleotides. The activity of *B. bacteriovorus* DNA polymerase III is not likely to be the rate-limiting factor, since the subunits of this holoenzyme exhibit high homology with those of *E. coli* (Makowski et al. 2019).

In binary-dividing *B. bacteriovorus*, after the initiation of DNA replication, the replication forks split; one fork remains at the invasive pole, while the other migrates toward the opposite pole. Both forks eventually move to the mid-cell, where the replication is terminated, resulting in the localization of both *ter* sites in this region of the cell (Fig. 3). Meanwhile, one of the newly replicated *oriC* regions migrates toward the former flagellar pole, which becomes an invasive pole. After cell division, two daughter cells are formed; each contains a compacted and polarized chromosome with the *oriC* region located at the invasive pole (Kaljević et al. 2021, Pląskowska et al. 2023) (Fig. 3).

When *B. bacteriovorus* divides through nonbinary fission, multiple replisomes (three or more) can be observed within a single filament (Figs 1 and 3) indicating that the next round of replication is (re)initiated before the previous round terminates. The duration of the S-phase is not directly proportional to the number of progeny cells: in filaments dividing into three or four daughter cells, e.g. the S-phases last ∼ 171 min or 187 min, respectively (Pląskowska et al. 2023). This further confirms the occurrence of overlapping replication rounds, i.e. multifork replication. The strategy of multifork replication not only appears in fast-growing unigenomic bacteria such as *E. coli* and *B. subtilis*, but has also been observed in slow-growing bacteria such as *Mycobacterium smegmatis* (Trojanowski et al. 2017) and multigenomic bacteria like *Corynebacterium glutamicum* (Böhm et al. 2017).

In HI mutants, the process of chromosome replication is supposed to be less strictly controlled; HI cells differ in size, shape, and presumably contain a different number of chromosome copies. However, to date, nothing is known about chromosome multiplication and septation within the filaments of HI mutants of *B. bacteriovorus*.

### Coordination of chromosome replication with chromosome segregation and septation

The coordination of key cell-cycle processes, such as that of chromosome replication with segregation and cell division, is crucial for the ability of a bacterial cell to produce viable offspring that contain the full complement of hereditary information. Bacteria have evolved various mechanisms to ensure that genetic information is faithfully transmitted to their offspring. These mechanisms are relatively well-known in model bacteria such as *E. coli, B. subtilis*, and *C. crescentus*, which divide by binary fission (Eswara and Ramamurthi 2017). However, less is known about the mechanisms that coordinate crucial cell cycle events in organisms dividing by nonbinary fission.

In bacteria, chromosome replication initiation must be closely coordinated with the segregation of one or more newly duplicated *oriC* regions. In most bacteria, this process is mediated by a ParAB*S* system consisting of ParA (an ATPase), ParB (binding to the *parS* sequence), and a centromere-like sequence called *parS* (Kawalek et al. 2023). The ParAB*S* system has been identified in *B. bacteriovorus* and other nonbinary dividing bacteria, including *Streptomyces* (Kim et al. 2000). In *B. bacteriovorus*, two *parS* sequences were recently identified near the *oriC* region (Fig. 2) and shown to be bound *in vitro* by the ParB protein (Kaljević et al. 2023). Microscopic observations revealed that shortly after the initiation of the first replication round, one of the newly synthesized *oriC* regions (visible as ParB complexes) is segregated to the former flagellar pole (Fig. 3) (Kaljević et al. 2021, Pląskowska et al. 2023). As chromosome multiplication progresses, the number of ParB segregation complexes, which corresponds to the number of released daughter cells, gradually increases as new copies of the *oriC* region are synthesized. Before septation, chromosomes must be compacted and uniformly distributed along the filament to ensure that each daughter cell receives a single copy of the chromosome. ParA is presumably necessary for the proper assembly of ParB complexes and, together with other yet-unidentified proteins, contributes to the precise positioning of the chromosome copies. However, the role of ParA in chromosome segregation has yet to be elucidated in *B. bacteriovorus*. Recent studies revealed that the three elements of the ParAB*S* system experience multilayered regulation at the levels of *parA* and *parB* gene transcription, ParA and ParB protein levels, and *parS* accessibility. Moreover, the ratio between ParA and ParB (ParA:ParB) fluctuates during the cell cycle and is presumed to be crucial both for the regulation of segrosome formation and for cell cycle progression (Kaljević et al. 2023).

At the end of the proliferation phase, *B. bacteriovorus* undergoes multiple synchronized divisions (Fenton et al. 2010) to convert a multichromosome filament into unigenomic daughter cells. In bacteria, the assembly of the division machinery is orchestrated by the FtsZ protein, a bacterial tubulin homolog that polymerizes into a Z-ring at each future site of cell division (Margolin 2005, Cameron and Margolin 2023). Investigation of the septation process revealed that two or more FtsZ rings are formed inside a single elongated cell during predation on large prey. Interestingly, the assembly of FtsZ rings was found to be asynchronous, in contrast to their synchronous disassembly. Notably, in both binary and nonbinary dividing cells, FtsZ ring assembly begins before DNA synthesis ends. In filaments undergoing nonbinary fission, the multiple FtsZ rings are assembled sequentially (Fig. 3) (Pląskowska et al. 2023). However, the factors responsible for determining the timing and positioning of Z-rings in *B. bacteriovorus* remain a mystery. In this bacterium, a replication checkpoint may act to coordinate chromosome multiplication with the cycle progression, such as by tuning FtsZ ring placement. In contrast to the situation in *B. bacteriovorus*, the FtsZ rings of *Streptomyces* are synchronously assembled (Grantcharova et al. 2005, Jakimowicz and van Wezel 2012) after DNA replication ends (Szafran et al. 2021).

Upon replication termination, daughter chromosomes must be physically separated and translocated before filament division is completed. Similar to the situation in other bacteria possessing circular chromosomes, dimeric chromosomes may form during DNA replication in *B. bacteriovorus* filaments. This problem is expected to be even more pronounced than in other bacteria, as within a single filament, multiple chromosomes are synthesized. In model organisms such as *E. coli* or *B. subtilis*, dimers are resolved by XerC and XerD, two tyrosine recombinases that target the 28-nucleotide motif (*dif*) associated with the chromosome’s replication terminus. In the *B. bacteriovorus* genome, the *dif* site (see Fig. 2) and *xerCD* genes (Bd3064, Bd2300) have also been identified through *in silico* analysis (Carnoy and Roten 2009). Finally, multiple chromosomes must be translocated away from the future septum sites to prevent them from becoming trapped or guillotined by a forming septum. In *B. bacteriovorus*, a DNA translocase called FtsK presumably coordinates chromosome translocation with cell division, as a *ftsK* gene (Bd0041) was identified in this bacterium (Heidari Tajabadi et al. 2017).

Bacteria have evolved both positive and negative mechanisms to spatially and temporally control the polymerization of FtsZ into Z-rings. The negative regulatory mechanisms include the Min system (in *E. coli* and *B. subtilis*) and nucleoid occlusion protection (in *E. coli* and *B. subtilis*), while the positive mechanisms include the PomXYZ (in *Myxococcus xanthus*) and MapZ (in *Streptococcus pneumoniae*) proteins (Mahone and Goley 2020). However, no such system has yet been identified in *B. bacteriovorus*. It is tempting to speculate that *B. bacteriovorus* uses one or more of its own mechanisms to precisely control the temporal and spatial placement of FtsZ rings. Such a mechanism would prevent the formation of offspring with an extra or missing/guillotined chromosome. We cannot exclude the possibility that one or more proteins that interact with ParA (or ParB) and/or contribute to cell division contribute to coordinating chromosome replication and segregation with filament septation. In *C. crescentus*, MipZ has been suggested to coordinate these processes by interacting with ParB at the cell poles and directly inhibiting the polymerization of FtsZ into Z-rings. MipZ forms a gradient within the cell, with its highest concentration at the poles and lowest at the midcell (Thanbichler and Shapiro 2006, Corrales-Guerrero et al. 2022).

## Conclusions and future directions

Although *B. bacteriovorus* was discovered over 60 years ago (Stolp and Petzold 1962, Stolp and Starr 1963), this predatory bacterium only relatively recently emerged as a novel and intriguing model organism. Due to its peculiar life cycle and bimodal proliferation (Figs 3 and 4), *B. bacteriovorus* represents a noncanonical yet attractive model for studying novel aspects of bacterial cell biology, including chromosome organization and the choreography of chromosome replication. In contrast to model organisms such as *E. coli* and *C. crescentus*, but similar to *Streptomyces*, this bacterium significantly changes its cell size during the life cycle (Fig. 4). Consequently, the chromosomes of *B. bacteriovorus* must be profoundly remodeled: they are highly compacted during the attack phase and septation, but become more relaxed during the proliferation phase. A recently identified histone (Bd0055) in this predatory bacterium appears to be a promising candidate for contributing to the efficient compaction of bacterial chromatin (Hocher et al. 2023). It is particularly noteworthy that the mechanism used by this novel bacterial histone to compact DNA differs from that used by eukaryotic histones (as mentioned earlier). It cannot be excluded that this protein, along with one or more other yet unidentified proteins, may help orchestrate the remodeling of bacterial chromatin. Further studies are needed to identify the proteins (including regulators) and mechanisms responsible for the extensive changes seen in chromosome organization during the life cycle of *B. bacteriovorus*. Chromosome decompaction begins upon the initiation of DNA replication (Kaljević et al. 2021), suggesting that a checkpoint may coordinate chromosome organization and replication with cell cycle progression.

Similarly to the situation in other asymmetric bacteria, such as *C. crescentus* (Fig. 4) and *V. cholerae*, the *oriC* region of *B. bacteriovorus* is located at a cell pole (the invasive pole). In this predatory bacterium, a region near the origin of replication must be anchored at the pole by a yet unknown protein complex. In nonbinary proliferating filaments, each subsequent round of chromosome replication is generally initiated from the invasive pole-anchored *oriC*; the exception to this is found in filaments dividing into five or more progeny cells, in which the opposite pole can becomes the invasive pole at a late stage of chromosome multiplication. Given this asynchronous mode of replication, the mechanisms responsible for controlling replication initiation are expected to be more intricate than those described for a model organism such as *E. coli*. Since the DNA replication of *B. bacteriovorus* starts specifically from the invasive pole-localized chromosome, it can be assumed that only the *oriC* attached to this cell pole is licensed for firing, while other *oriC* regions remain replicative silent (“dormant”). Similarly, in eukaryotes, only licensed origins can initiate replication (Marks et al. 2017). Thus, in *B. bacteriovorus*, the initiation of chromosome replication is both temporarily and spatially regulated. These unique features of replication initiation choreography raise interesting questions, such as: (1) Is there an *oriC* licensing system? (2) Why do potential replication *oriC* regions remain inactive? (3) What mechanisms control the licensing and dormancy of *oriC* regions? and (4) How are *oriC* licensing and DNA replication coordinated/regulated during the proliferation of this predatory bacterium?

Many other interesting questions remain open regarding the cell biology of *B. bacteriovorus*. For example, we need to understand when and how the predatory cell prevents the next DNA replication round from being triggered in advance, such that all cell cycle events are finished before the available prey resources are completely exploited. The HI mutant of *B. bacteriovorus* is also a challenging model, and further studies are needed to delineate how the chromosomes of HI mutants are organized and how these mutants replicate.
